# Native fluorescent detection with sequential injection chromatography for doping control analysis

**DOI:** 10.1186/1752-153X-7-144

**Published:** 2013-08-28

**Authors:** Abubakr M Idris, Ahmed O Alnajjar

**Affiliations:** 1 Department of Chemistry, College of Science, King Khalid University, P.O. Box 9004, Abha, Saudi Arabia; 2 Department of Chemistry, College of Science, King Faisal University, Hofuf, Saudi Arabia

**Keywords:** Sequential injection chromatography, High performance liquid chromatography, Amiloride, Furosemide, Method validation

## Abstract

**Background:**

Sequential injection chromatography (SIC) is a young, ten years old, separation technique. It was proposed with the benefits of reagent-saving, rapid analysis, system miniaturization and simplicity. SIC with UV detection has proven to be efficient mostly for pharmaceutical analysis. In the current study, a stand-alone multi-wavelength fluorescence (FL) detector was coupled to an SIC system. The hyphenation was exploited for developing an SIC-FL method for the separation and quantification of amiloride (AML) and furosemide (FSM) in human urine and tablet formulation.

**Results:**

AML and FSM were detected using excitation maxima at 380 and 270 nm, respectively, and emission maxima at 413 and 470 nm, respectively. The separation was accomplished in less than 2.0 min into a C18 monolithic column (50 × 4.6 nm) with a mobile phase containing 25 mmol/L phosphate buffer (pH 4.0): acetonitrile: (35:65, v/v). The detection limits were found to be 12 and 470 ng/mL for AML and FSM, respectively.

**Conclusions:**

The proposed SIC-FL method features satisfactory sensitivity for AML and FSM in urine samples for the minimum required performance limits recommended by the World Anti-Doping Agency, besides a downscaled consumption of reagents and high rapidity for industrial-scale analysis of pharmaceutical preparations.

## Background

**Figure 1:**
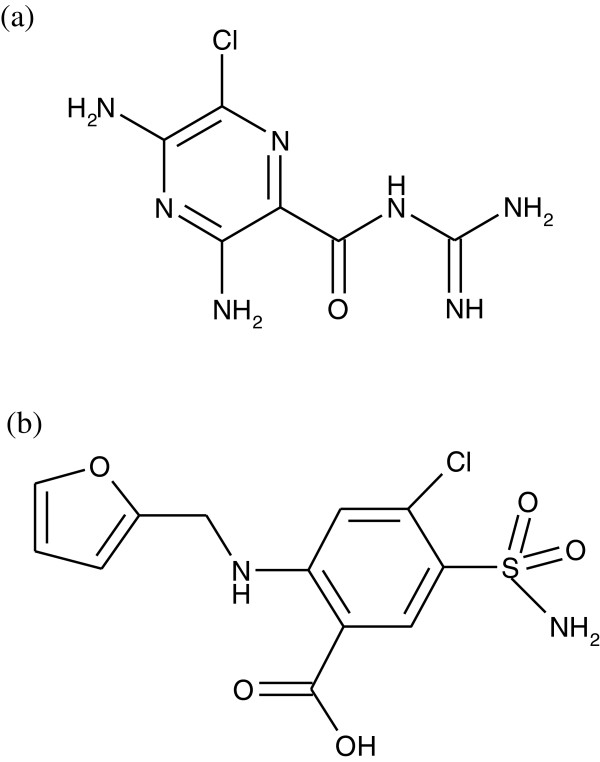
Chemical structures of (a) amiloride and (b) furosemide.

Furosemide (FSM) is a loop diuretic. It is an anthranilic acid derivative, which is chemically 4-chloro-N-furfuryl-5-sulfamoylanthranilic acid (Figure 1a). FSM acts inhibiting the co-transportation of sodium, potassium and chloride. It further causes the excretion of calcium, magnesium and bicarbonate ions [1,2]. In another context, amiloride (AML) is chemically N-amidino-3,5-diamino-6-chloropyrazine-2-carboxamide (Figure 1b). AML, as another potent loop diuretic, acts primarily by blocking sodium and chloride reabsorption in the ascending limb of the loop of Henle. FSM helps to conserve potassium and minimize the risk of alkalosis. It is also used in the treatment of oedema associated with hepatic cirrhosis and congestive heart failure [3,4].

The association of AML and FSM furnishes a valuable natriuretic agent with a diminished kaliuretic effect and minimizes the risk of alkalosis in the treatment of refractory oedema associated with hepatic cirrhosis or congestive heart failure [5]. Due to the benefits of their simultaneous use, AML and FSM are being prepared as binary dosage forms. Accordingly, the development of assay methods for those two drugs is desirable for the purpose of quality control. In this issue, various analytical techniques were exploited including high performance liquid chromatography [4,6,7], spectrophotometry [8-10], fluorometry [2] and electroanalytical [11].

On the other hand, athletes use diuretics, in general, for flushing previously taken prohibited substances with forced diuresis [12] to achieve acute weight loss. Hence, the World Anti-Doping Agency (WADA) prohibits the use of diuretics [13]. Besides being an ethically condemned practice, the risk to the athletes’ health has to be considered since they are generally self-administered in a wrong manner; i.e. overdoses, interactions with other drugs or even the use of drugs of illicit origin [14-16]. Evidently, a sensitive and reliable analytical method to determine diuretics in urine and/or plasma is a prerequisite in sport activities. Toward this end, WADA establishes a minimum detection capability for testing methods called the Minimum Required Performance Limits (MRPL). This is to ensure that all doping control laboratories can report the presence of prohibited substances uniformly. The limit for each analyte in the class of diuretics is 250 ng/mL [17,18].

The dominant techniques used for screening diuretics in control urinalysis are GC and HPLC. However, both techniques have the limitations of the high cost of instrumentation and maintenance. Moreover, other challenges in GC namely are the low volatility of the compounds and the necessity of the additional step of derivatization. HPLC has also the limitation of large consumption of solvent volumes, which is due to the continuous flow of mobile phase and large instrumentation dimension.

Recently, sequential injection chromatography (SIC) was introduced to overcome some challenges in separation techniques [19]. In principle, the procedure of SIC is based on a sequential injection, i.e. a discontinuous-flow approach, of a mobile phase and samples. The separation process is usually carried out into a monolith column using programmable miniaturized modules. The association of the three approaches of the discontinuous-flow approach, monolith separation column and system miniaturization renders SIC procedure simple, rapid and reagent-saving [20-22]. On the other side, the major limitation of SIC is the limited pressure of the syringe pump. The maximum is 900 psi. This causes back-pressure in separation column and hence limits the use of long separation column and hence reduces the separation capability of many analytes. However, nineteen chromatographic peaks for amino acids were observed in an SIC profile in a previous study [23]. Another limitation of SIC is that the limited volume of the syringe. The commercially available syringe volume is 10 mL, which might not be sufficient for eluting all compounds from a separation column. However, this problem could be solved by refilling the syringe.

In the current study, a SIC system was coupled with a fluorescence (FL) detector to provide an analytical method for the separation and quantification of AML and FSM in human urine and pharmaceutical formulation. The capabilities of that couple were exploited in terms of reagent-saving, analysis time, sensitivity and selectivity.

## Results and discussion

### Method optimization

A short monolith column (50 × 4.6 mm) was examined and, initially, it has been found to be sufficient for the separation of the two drugs. With respect to column dimension, the practicable flow rate of 20–40 μL/s in SIC was tested [24-30]. It is well known that high flow rate accelerates analysis and sharpens peaks. In contrast, high flow rates increases the back-pressure in a separation column. Accordingly, the optimum flow rate set in the current study was 25 μL/s. On the other hand, with respect to peak height and peak shape, the practicable range of sample volume is 40–60 μL [24-30]. At a large sample volume, peak height was significantly improved while acceptable peak shape was not achieved. Hence, the optimum sample volume has been found to be 30 μL.

**Figure 2:**
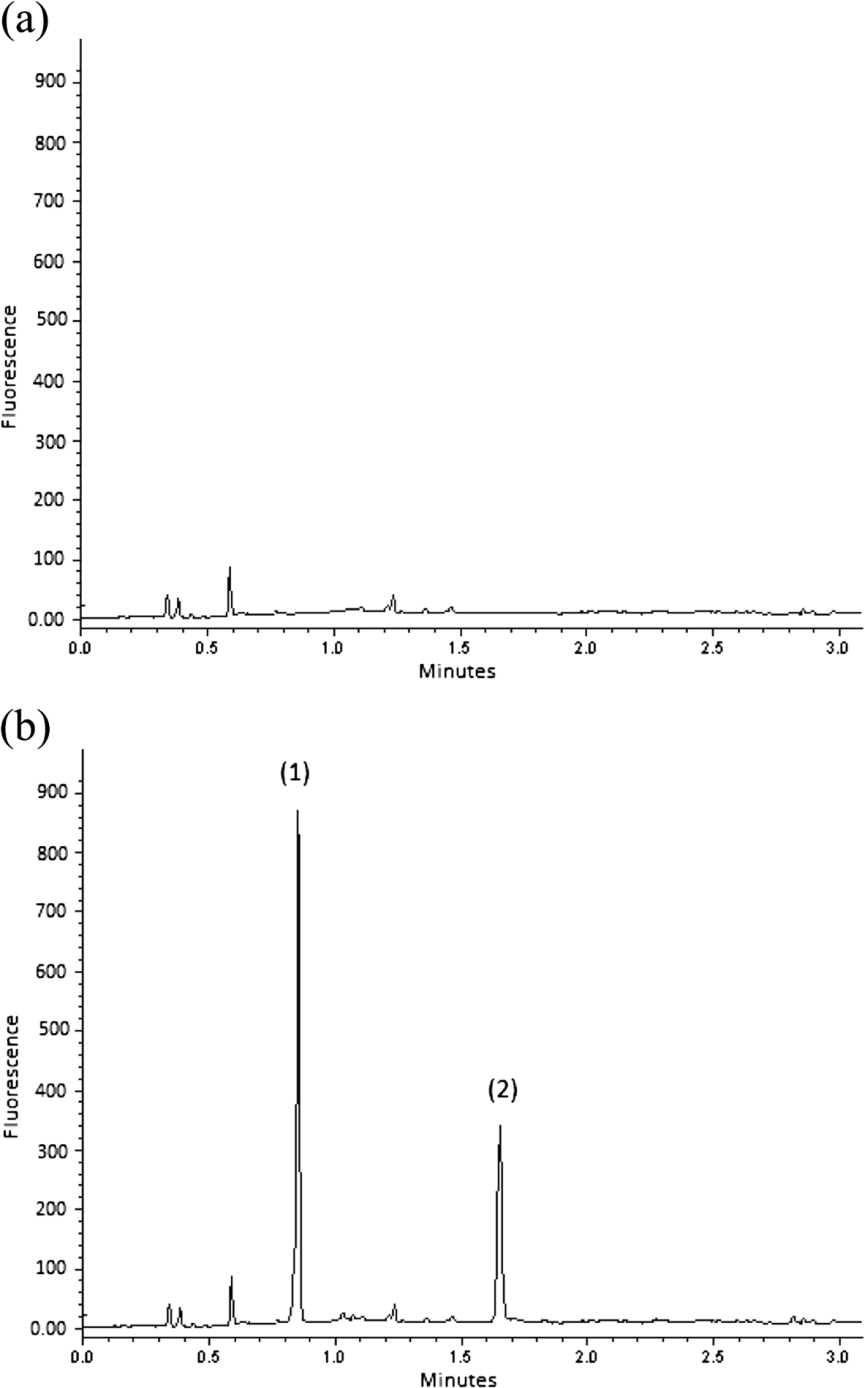
SIC profiles following fluorescence of: (a) urine sample after solid-phase extraction and (b) urine sample spiked with (1) amiloride and (2) furosemide to get concentration of 3.0 μg/mL of each drug.

For the optimization of mobile phase composition, acetonitrile, primarily, shows better separation than methanol. Moreover, phosphate buffer was found be essential for satisfactory separation. The preliminary study indicated the possible ranges of pH, buffer concentration and the percentage volume of acetonitrile (Table 1). The 2^3^ full-factorial design was adopted to screen the effect of those three conditions on resolution, retention time and peak area. UV detection at 380 and 270 nm for AML and FSM, respectively, was adopted for optimization. Thereafter, the optimum conditions were applied for fluorescence detection for further studies, namely method validation and application. The results obtained are compiled in Table 2. More than sufficient separation with a resolution of ≥ 4.5 was obtained at high percentage volume of acetonitrile and different levels of pH and buffer concentration. At those conditions, the retained FSM was eluted at 0.94 to 2.43 min. Also, relatively, large peak area of both drugs was obtained at those conditions. In contrast, low percentage volume of acetonitrile, with either high level or low level of pH and buffer concentration, did not exhibit sufficient separation. Table 2 shows the conditions and results of three additional experiments, which were carried out with fixing pH and buffer concentration at their high levels. Table 2 shows that the mobile phase composition of 25 mmol/L phosphate: acetonitrile (35:65, v/v) at pH 4.0 achieved more than sufficient separation with a resolution of 8.2 and short retention times of both AML and FSM. Additionally, fully symmetrical peak of AML (1.00) and semi-symmetrical peak of FSM (0.89) were achieved (Figure 2). Eventually, Table 3 presents the optimum instrumental and chemical conditions proposed to the current SIC-FL method.

**Table 1:** **Minimum and maximum levels of pH, buffer concentration and percentage volume of acetonitrile adopted for the 2**^**3 **^**full-factorial design matrix for method optimization**

**Condition**	**Minimum**	**Maximum**
pH	3.0	4.0
Buffer concentration (mmol/L)	10	25
Percentage volume of acetonitrile (%)	30	70

**Table 2:** **2**^**3 **^**full-factorial design matrix**^**a **^**and further experiments**^**b **^**for screening the effect of pH buffer concentration and percentage volume of acetonitrile on resolution (R), retention time (t**_**R**_**, min) and peak area**

**pH**	**BC**^**a**^	**ACN%**	**R**	**t**_**R**_		**Peak area**	
				**AML**	**FSM**	**AML**	**FSM**
4.0	25	30	< 1.0	-	-	-	-
4.0	25	70	8.5	0.89	0.95	975161	990962
4.0	10	30	< 1.0	-	-	-	-
4.0	10	70	8.5	0.89	0.94	986511	981150
3.0	25	30	< 1.0	-	-	-	-
3.0	25	70	8.7	2.25	2.37	2908429	6670422
3.0	10	30	< 1.0	-	-	-	-
3.0	10	70	8.2	2.35	2.43	931563	1070808
4.0	25	50	6.9	0.78	1.06	1044527	1072148
4.0	25	60	7.8	0.84	1.20	1007888	750986
4.0	25	65	8.2	0.85	1.62	1032450	901919

^a^the first eight rows compile the 2^3^ full-factorial design matrix; ^b^the last three rows compile further experiments.

**Table 3:** Optimal analytical conditions of the SIC-FL method

**Analytical character**	**AML**	**FSM**
Separation column	(50 × 4.6 mm)	
Mobile phase composition	25 mm phosphate : acetonitrile (35:65 v/v, pH 4.0)	
Sample volume (μL)	30	
Flow rate (μL/s)	25	
Excitation wavelength (nm)	380	270
Emission wavelength (nm)	413	470

### Method validation

The validation metrics including linearity, recovery and simple and complex precision as well as the limits of detection and quantitation were determined from measurements.

For linearity studies, samples were prepared using the highest calibrators, namely 100 μg/mL AML and 200 μg/mL FSM. Serial dilutions were made to achieve AML concentrations of: 0.1, 0.5, 1.0, 2.0, 3.0 and 4.0 μg/mL; and FSM concentrations of: 2.0, 3.0, 4.0, 6.0, 8.0 and 10.0 μg/mL. Linearity was determined via the least squares linear regression analysis of the data obtained from the average of three replicates from each of the levels described above. A previous spectrofluorimetric method [5] reported linear ranges of 3.7 × 10^-4^-0.8 and 1.2 × 10^-3^-4.0 μg/mL for AML and FSM, respectively. Despite these ranges are lower than the corresponding of the current SIC-FL method, the latter are sufficient for drug detection in urine and more suitable for pharmaceutical analysis.

Simple (within-run) precision was evaluated through the analysis of three spiked urine samples, which were run seven times. The first sample included 0.02 AMLμg/mL and 0.40 FSMμg/mL. The second sample included 0.03 AMLμg/mL and 0.80 FSMμg/mL. The third sample included 0.40 AMLμg/mL and 1.20 FSMμg/mL. The samples were injected into the SIC-FL system after SPE with a preconcentration factor of 5. Mean and RSD were assessed at each level. Complex (between-run) precision for the method was determined through the analysis of the aforementioned samples injected once per day for five days. Once again, mean and RSD were evaluated. In general, Table 4 shows acceptable precision, at its two levels simple and complex, for both AML and FSM quantification.

**Table 4:** Validation metrics results of the SIC-FL method

**Analytical character**	**Result**	
Resolution	8.2	
Consumed mobile phase volume (mL)	4.5	
Total analysis time (min)	4.7	
Sample frequency (samples/h)	13	
	AML	FSM
Retention time (min)	0.85	1.62
Peak symmetry	1.00	0.89
Theoretical plates	671.55	937.95
Regression equation	PA^a^ = 1.6387C^b^ + 566.28	PA^a^ = 26.667C^b^ + 23.333
Correlation coefficient	0.9971	0.9987
Linear range (μg/mL)	0.1-4.0	2.0-10.0
Within-run precision (RSD, %)	1.98	2.02
Between-run precision (RSD, %)	0.57	2.81
Recovery in urine samples (%)	89	91
Recovery in tablets (%)	99.1 for 1.0 μg/mL	98.7 for 8.0 μg/mL
	97.5 for 0.5 μg/mL	98.1 for 4.0 μg/mL
	97.0 for 0.5 μg/mL	96.5 for 0.5 μg/mL
LOD (μg/mL)	0.012	0.470
LOQ (μg/mL)	0.060	1.500

^a^peak area; ^b^concentration (μg/mL)

The LOD was determined at a signal-to-noise ratio of 3 whereas the LOQ was determined at a signal-to-noise ratio of 10. The noise was assessed using drug-free human urine samples after SPE. Interestingly, the LOQs of AML and FSM (Table 4) achieved from the current SIC-FL match the MRPL that was recommended by WADA [17,18]. A previous HPLC screening test was presented for some diuretics of doping interest in human urine [16]. In that method, the LOD for AML and FSM were 0.750 and 0.125 μg/mL, respectively. These levels are not in consistence with the current SIC-FL method. This could be attributed to the use of liquid-liquid extraction for sample treatment and UV for the detection in the previous HPLC method [16] while SPE and fluorescence detection were used in the current SIC method.

### Method application

The proposed SIC-FL method was applied to four urine samples. All samples were subjected to SPE. One sample was free from AML and FSM in order to examine the efficiency of SPE for sample clean-up and to calculate the LODs and LOQs. The chromatogram of one sample as an example is depicted in Figure 2a. Acceptable baseline was obtained indicating acceptable efficiency of the sample clean-up step in the adopted SPE procedure [31]. The other samples were spiked with different quantities of AML and FSM as described in the subsection entitled “Method validation”. As an example, Figure 2b shows the chromatogram of 3.0 μg/mL AML and FSM in a urine sample. The pre-concentration factor obtained from SPE was 5. Hence, the level of AML and FSM injected into the SIC-FL system was 0.6 μg/mL. The recovery obtained (Table 4) was acceptable indicating the accuracy of the SPE procedure [31] and the proposed SIC-FL method.

**Figure 3:**
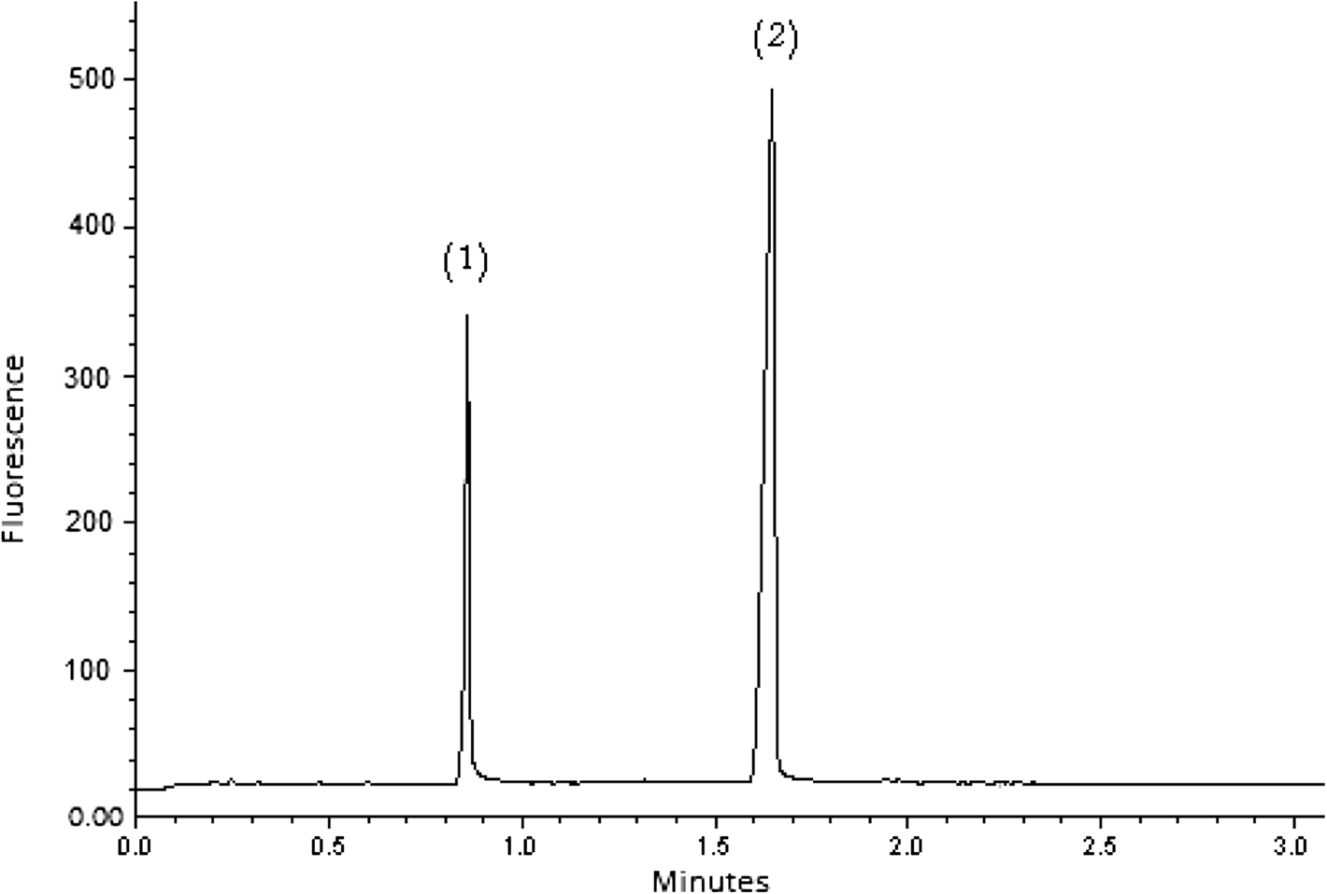
SIC profile following fluorescence of (1) 0.5 μg/mL AML and (2) 4.0 μg/mL FSM extracted from Frumil® Tablets.

The SIC-FL method was also applied to tablet formulation using three levels of AML and FSM as discussed in the section entitled “Preparation of reagents and samples”. As an example, Figure 3 shows an SIC profile of 0.5 μg/mL AML and 4.0 μg/mL FSM extracted from Frumil® Tablets. In general, the chromatograms obtained indicate the applicability of the SIC-FL method to the separation and quantification of AML and FSM to both urine samples and tablet formulation.

### Unique SIC analytical features

One of the interesting advantages of the proposed SIC-FL method over HPLC methods is mainly the reagent-consumption. The total volume of the consumed reagent, i.e. mobile phase for column conditioning and separation, was 4.5 mL. Hence, in routine analysis, SIC consumes milliters per day versus centiliters per day for HPLC. As previously mentioned, the reduction of reagent consumption in SIC is due to the discontinuous-flow, downscaled-dimension of instrumentation and the use of monolithic column. Accordingly, the waste production of SIC is less than that of HPLC and hence the frontal is greener than the latter. On the other side, the use of monolithic column and the miniaturization of SIC work hand in hand to provide a rapid analysis. The total analysis time including column conditioning and elution, without SPE, was 4.7 min. Hence, the sample frequency was 13 samples/h. Furthermore, the instrumentation simplicity of SIC offers less instrumentation cost. In addition, the simplicity in SIC instrumentation makes its maintenance cost less.

## Experimental

### Instrumentation

**Figure 4:**
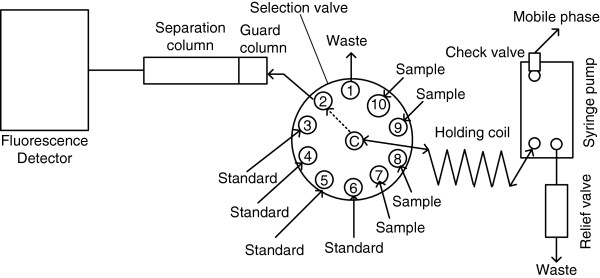
Schematic diagram of sequential injection chromatograph coupled with fluorescence detector constructed for the separation and quantification of amiloride and furosemide.

The SIC system used in the current study was SIChrom®. It was assembled by FIALab® (Medina, WA US). The SIC system (Figure 4) included syringe pump (SP), selection valve (SV), and tubings. The SV was 10 T-0179H Cheminert® high-pressure stainless-steel (up to 5000 psi) with 10 ports. It was manufactured by Valco Instrument Co. (Houston, TX, US). The SP was S17 PDP® with a reservoir of 4 mL. It was manufactured by Sapphire Engineering (Pocasset, MA, US). Pump tubing of 0.03" I.D. Teflon type was supplied from Upchurch Scientific, Inc. (Oak Harbor, WA, USA). It was used to connect various devices of the SIC manifold and to make a holing coil (200 cm long). (vi) PC equipped with FIALab Software® for Windows® version 5.9 was supplied from FIAlab (Medina, WA, USA).

The detector was 2475 Multi λ Florescence Detector from Waters (Milford, CT, US). The light source was a xenon lamb with excitation wavelength in the range of 200–900 nm. The detector was equipped with excitation and emission monochromators.

The solid phase extraction (SPE) columns of 6-mL size, which were used for urine sample treatment, were Discovery DSC-18. They were supplied by Supleco (Bellefonte, PA, US).

### Chemicals, reagents and samples

Double-distilled deionized water was used throughout the experimental work. All chemicals and reagents were of analytical reagent grade. AML hydrochloride hydrate, FSM, acetonitrile, sodium hydrogen phosphate, ortho-phosphoric acid were purchased from Sigma-Aldrich (Taufkirchen, Germany).

### Preparation of reagents and samples

100 μg/mL of AML and 200 μg/mL of FSM as stock standard solutions were prepared by dissolving appropriate amounts in mobile phase. Working standard solutions were prepared by dilution in an appropriate way.

10 mL of urine samples were obtained from eight healthy volunteers. All samples were filtered through Whatman® paper No 1. Two samples were then subjected directly to SPE without spiking drugs. The other samples were spiked in duplicate with three concentrations of each drug as described in the subsection entitled “Method validation”. Thereafter, the three replicate samples were subjected to SPE. 2.5 mL of urine were cleaned through the SPE columns by 3 mL of water and eluted with 2 mL of methanol. The methanolic extract was evaporated to dryness under nitrogen stream and reconstituted in 2 mL mobile phase. Blank urine was treated in same manner [31].

Frumil® tablets (5 mg of AML hydrochloride and 40 mg of FSM), which were prepared by Sanofi-Aventis (Dublin, Ireland), were examined. Twenty tablets were accurately weighed and finely powdered. Three portions were accurately weighed and transferred into 100-mL calibrated flasks. The first portion was equivalent to 5 mg of AML and 40 mg of FSM. The second portion was equivalent to 2.5 mg of AML and 20 mg of FSM. The third portion was equivalent to 1.25 mg of AML and 10 mg of FSM. The drugs were extracted by the mobile phase with shaking and filtration. The solutions were diluted fifty-times. The recovery in tablet formulation was examined using a previous HPLC method as a reference [4].

### SIC procedure

A rapid protocol controlling the proposed SIC procedure was programmed. 1.0 mL of the mobile phase was aspirated through the check valve in the syringe pump at a flow rate of 150 μL/s. For column conditioning, the mobile phase was introduced into the separation column through port-2 and the guard column (Figure 4) at a flow rate of 30 μL/s. The syringe was filled again with 3500 μL of the mobile phase at a flow rate of 150 μL/s. 30 μL of standards/samples were loaded into the holding coil through port-3 to port-10 at a flow rate of 10 μL/s. The sample and mobile phase were then injected into the guard and separation columns through port-2 at a flow rate of 25 μL/s. During this step, the fluorescence detector was set at excitation and emission wavelengths as presented in Table 3.

## Conclusions

A fluorescence detector was hyphenated to an SIC system to generate a sensitive and direct method for the separation and quantification of AML and FSM. The hyphenation also permitted a simple, inexpensive, rapid and reagent-saving procedure. The SIC-FL method was validated and it demonstrated to be reliable for the determination of both drugs, being linear, accurate and precise. Therefore, the SIC-FL method can be considered suitable for the quantification of both drugs in human urine samples. The SIC-FL method is also thought to be ideally suited for a rapid routine analysis for the quality control of pharmaceutical products.

## Competing interests

The authors declare that they have no competing interests.

## Authors' contributions

AMI proposed the design of the study and drafted the manuscript. AOA carried out the hyphenation of SIC-FL and other bench-works. Both authors read and approved the final manuscript.

## Authors’ information

Dr. Abubakr M. Idris is a Sudanese associate professor of analytical chemistry at the Department of Chemistry, College of Science, King Khalid University, Abha, Saudi Arabia. Idris has authored more than sixty-five papers and books published in international refereed journals and conferences. His research focuses on developing microfluidic analytical technologies and their methodologies. He has some publications on environmental issues as well. Idris joints the editorial board of American Journal of Analytical Chemistry, Development in Analytical Chemistry and Novus Scientia Journals. He also gained awards for funding more than fifteen research projects from various institutes. On the other hand, Idris has many activities on the issues of academic development and quality. He is currently the chairman of the Unit of Academic Development and Quality, College of Science, King Khalid University.
